# Prediction of non-small cell lung cancer subtypes is possible through restricted spectrum imaging

**DOI:** 10.3389/fonc.2025.1737182

**Published:** 2026-01-19

**Authors:** Lei Shen, Yipin Zhang, Zhun Huang, Bo Dai, Yang Yang, Zhe Wang, Xuan Yu, Nan Meng, Fang Fang Fu

**Affiliations:** 1 Department of Radiology, Henan Provincial People’s Hospital and Zhengzhou University People’s Hospital, Zhengzhou, China; 2 North Henan Medical University, Xinxiang, China; 3 Beijing United Imaging Healthcare Co., Ltd., Beijing, China; 4 Central Research Institute, United Imaging Healthcare Group, Shanghai, China

**Keywords:** diffusion-weighted imaging, non-small cell lung cancer, quantitative imaging, restricted spectrum imaging, subtypes

## Abstract

**Background:**

To evaluate the utility of restricted spectrum imaging (RSI) for predicting subtypes of non-small cell lung cancer (NSCLC).

**Methods:**

A total of 97 patients with NSCLC (30 with squamous cell carcinoma (SCC) and 67 with adenocarcinoma (AC)) were included. The parameters f_1_, f_2_, f_3_, apparent diffusion coefficient (ADC), and maximum standardized uptake value (SUV_max_) were measured and compared between the two subtypes. Logistic regression analysis was used to identify independent predictors, and a combined diagnostic model was developed. The performance of the model was assessed using receiver operating characteristic (ROC) curve analysis, calibration curves, and decision curve analysis (DCA).

**Results:**

Compared with the AC group, the SCC group exhibited significantly higher SUV_max_, f_2_, and f_3_ values, and lower ADC and f_1_ values (all P < 0.05). Smoking status, f_1_, SUV_max_, and ADC were independent predictors of NSCLC subtypes. The combined model demonstrated superior diagnostic accuracy (AUC = 0.909; sensitivity = 73.33%; specificity = 89.55%) compared with individual predictors (AUC = 0.693, 0.819, 0.767, and 0.742 for smoking status, f_1_, SUV_max_, and ADC, respectively; all P < 0.01). Bootstrap resampling (1000 samples) validated the robustness of the model (AUC = 0.895). Calibration curves and DCA confirmed the model’s stability and clinical utility.

**Conclusion:**

RSI can effectively differentiate NSCLC subtypes.

## Introduction

Non-small cell lung cancer (NSCLC) is one of the most fatal malignancies globally (1). Squamous cell carcinoma (SCC) and adenocarcinoma (AC) are both prevalent histological subtypes of NSCLC, but their diagnosis and prognosis differ considerably (2). For example, in terms of surgical procedures, SCC may require more extensive airway reconstruction, whereas AC surgery is less invasive; in terms of drug therapy, patients with AC are more suitable for targeted therapy, while those with SCC are more suitable for immunotherapy. Additionally, SCC and AC differ in the assessment of recurrence risk and drug resistance (3–5). Therefore, accurate assessment of NSCLC subtypes before treatment is of great significance for the development of personalized treatment plans in clinical practice.

Although image-guided biopsy and bronchoscopy remain the gold standards for NSCLC subtype identification, they are invasive and pose risks such as bleeding and pneumothorax (6). Advances in quantitative imaging techniques have provided noninvasive alternatives for tumor characterization. Diffusion-weighted imaging (DWI), which assesses the diffusion of water molecules within tissues, and ¹^18^F-fluorodeoxyglucose positron emission tomography (^18^F-FDG PET), which evaluates tumor metabolism, are widely used for lung cancer assessment (7, 8). Restricted spectrum imaging (RSI) is an advanced diffusion MRI model that improves on conventional DWI by distinguishing water diffusion into restricted, hindered, and free compartments through a linear combination of diffusion-weighted models (9, 10). This enables RSI to quantitatively characterize the movement of water molecules in biological tissues with greater precision. RSI has been preliminarily applied in tumor evaluation. For instance, a study by Krishnan et al. showed that RSI helped improve the risk stratification of patients with glioblastoma (11); a study by Zhang et al. found that RSI-derived metrics could be used to noninvasively and effectively identify microvascular invasion in hepatocellular carcinoma (12); and a breast-related study conducted by He et al. concluded that RSI was able to quantitatively characterize breast lesions and normal fibroglandular tissue (13). However, in lung cancer research, to the best of our knowledge, only a few studies have explored the value of RSI in identifying benign and malignant lesions (14).

This study aims to evaluate the diagnostic value of RSI-derived quantitative parameters in differentiating SCC from AC, compare these parameters with classical ^18^F-FDG PET and DWI metrics, and combine them to develop a diagnostic tool. The ultimate goal is to provide a novel reference for the noninvasive assessment of NSCLC subtypes.

## Materials and methods

### Population

This research was approved by the local ethics review board, and all participants provided written informed consent. From June 2021 to October 2025, a total of 142 patients suspected of having lung cancer based on clinical evaluation or CT imaging underwent chest multiparametric scanning. The exclusion criteria were as follows: 1) Patients with claustrophobia or other conditions that prevented the completion of all imaging sequences (n = 8); 2) Scans with poor image quality (e.g., significant artifacts) that made them unsuitable for analysis (n = 14); 3) Cases where the interval between scanning and biopsy exceeded two weeks (n = 10); 4) Histological findings that did not indicate SCC or AC (n = 7); and 5) Patients who had received relevant treatment before scanning (n = 6). After applying these criteria, 97 patients were included in the study. Baseline characteristics such as age, sex, smoking status, and tumor size were recorded.

### Scanning protocols

The MRI sequences (3.0 T system, uPMR790, United Imaging, Shanghai, China) included axial T2-weighted imaging (T2WI) and DWI with multiple b-values. The ^18^F-FDG used in this study was sourced from FracerLab FX-FDG (GE Minitrac), with a purity > 95% and a pH range of 4.5–8.5. Patients fasted for at least 6 hours to ensure their serum glucose levels remained ≤ 6.5 mmol/L before receiving an ^18^F-FDG injection (0.11 mCi/kg). The PET scan began 60 minutes after injection, covering the upper thoracic inlet to the lower lung margin with the patient in the supine position (15, 16). PET image reconstruction was performed using the ordered subset expectation maximization (OSEM) method (2 iterations, 20 subsets, voxel size 2.6 × 2.6 × 2.0 mm³). A detailed summary of the protocol specifications is provided in **Table 1**.

**Table 1:** Scanning protocol.

Parameters	T2WI	Multiple b-Value DWI
Sequence	Axial FSE	Axial SS - EPI
Fat suppression	Yes	Yes
TR/TE (ms)	3315/87.8	1620/69.6
Respiratory compensation	Yes	Yes
FOV (cm^2^)	35 × 50	35 × 50
Bandwidth (Hz/pixel)	260	2370
Matrix	264 × 480	202 × 256
Slice thickness/Interval (mm)	5/1	5/1
Number of excitations	2	1, 1, 2, 2, 4, 4, 6, 6, 8, 10, 10, 10
b-values (s/mm^2^)	/	0, 25, 50, 100, 150, 200, 400, 600, 800, 1000, 1500, 2000
Scan time	2 min 26 s	4 min 22 s

T2WI, T2-weighted imaging; DWI, diffusion-weighted imaging; FSE, fast spin echo; SS-EPI, single shot echo planar imaging; TR/TE, repetition time/echo time; FOV, field of view.

### Parameter generation

The acquired images were transferred to a post-processing workstation (uWS-MR005, United Imaging, Shanghai, China) for registration, motion correction, and in-depth analysis. DWI and RSI data were processed using diffusion analysis software from the Advanced Analysis Toolkit. The DWI parametric pseudo-color map was generated using Equation 1:

(1) $${\text{S}}_{\text{b}}/{\text{S}}_{0}=\;\exp\;\left(-\text{ b}\times \mathrm{ADC}\right)$$

where ADC represents the apparent diffusion coefficient, b is the diffusion sensitizing factor, and S_0_ and S_b_ denote the signal intensities (SIs) at b = 0 s/mm² and b = [specified value] s/mm², respectively (8). The RSI parametric pseudo-color map was constructed using Equation 2:

(2) $$S(b)=f_{1}e^{-bD1}+f_{2}e^{-bD2}+f_{3}e^{-bD3},D1<D2<D3$$

where f_1_, f_2_, and f_3_ represent the volume fractions of the restricted, hindered, and free water diffusion compartments, respectively, and D1, D2, and D3 denote the ADCs of these compartments. To prevent overfitting, ensure model linearization, and maintain comparability across compartments, D1, D2, and D3 were globally assigned values of 1.0 × 10^-3^ mm²/s, 2.0 × 10^-3^ mm²/s, and 3.0 × 10^-3^ mm²/s, respectively, based on established theoretical values and experimental data (17, 18).

Tumor margins on axial T2-weighted images were manually delineated slice by slice to define regions of interest (ROIs). Lesions with cystic degeneration, necrosis, hemorrhagic artifacts, or blood vessels were excluded. The finalized ROIs were then mapped onto pseudo-color DWI and RSI parameter maps, and the mean values were extracted. The volume of interest (VOI) was automatically delineated, and the maximum standardized uptake value (SUV_max_) was calculated using PET fusion software. Two radiologists performed these procedures independently: an attending radiologist with 8 years of experience and an associate chief radiologist with 15 years of experience. Both were blinded to each other’s results and the patients’ clinicopathological details.

### Histopathologic assessment

Tumor specimens were obtained through surgical resection or biopsy. Histological subtype classification was performed in accordance with the guidelines of the International Association for the Study of Lung Cancer (IASLC) (19).

### Data analysis

We employed R (version 3.5.3, R Foundation, Auckland, New Zealand) and SPSS (version 27.0, MedCalc Software, Ostend, Belgium) to conduct data analysis. To assess the interobserver agreement for the parameters, we utilized the interclass correlation coefficient (ICC). An ICC > 0.75 was considered indicative of satisfactory reliability (20). Based on the characteristics of the variables, different statistical tests were applied to compare data between the SCC and AC groups. These tests included the Mann–Whitney U test, independent samples t - test and chi - square test.

R (version 3.5.3, R Foundation, Auckland, New Zealand) and SPSS (version 27.0, IBM Corp., Armonk, NY, USA) were used for data analysis. The interclass correlation coefficient (ICC) was employed to assess the interobserver agreement for the parameters. An ICC > 0.75 was considered indicative of satisfactory reliability (20). Based on the characteristics of the variables, different statistical tests were applied to compare data between the SCC and AC groups, including the Mann–Whitney U test, independent samples t-test, and chi-square test. The diagnostic performance of the parameters was assessed using the area under the receiver operating characteristic curve (AUC). The DeLong test was used to compare differences in AUC values. Logistic regression (LR) analysis was performed to identify independent predictors and develop a multiparameter composite diagnostic tool. Bootstrap resampling (1000 samples), calibration curves, and decision curve analysis (DCA) were used for internal validation and evaluation of the diagnostic tool. Statistical significance was set at P < 0.05.

## Results

### Baseline characteristics

**Figure 1:**
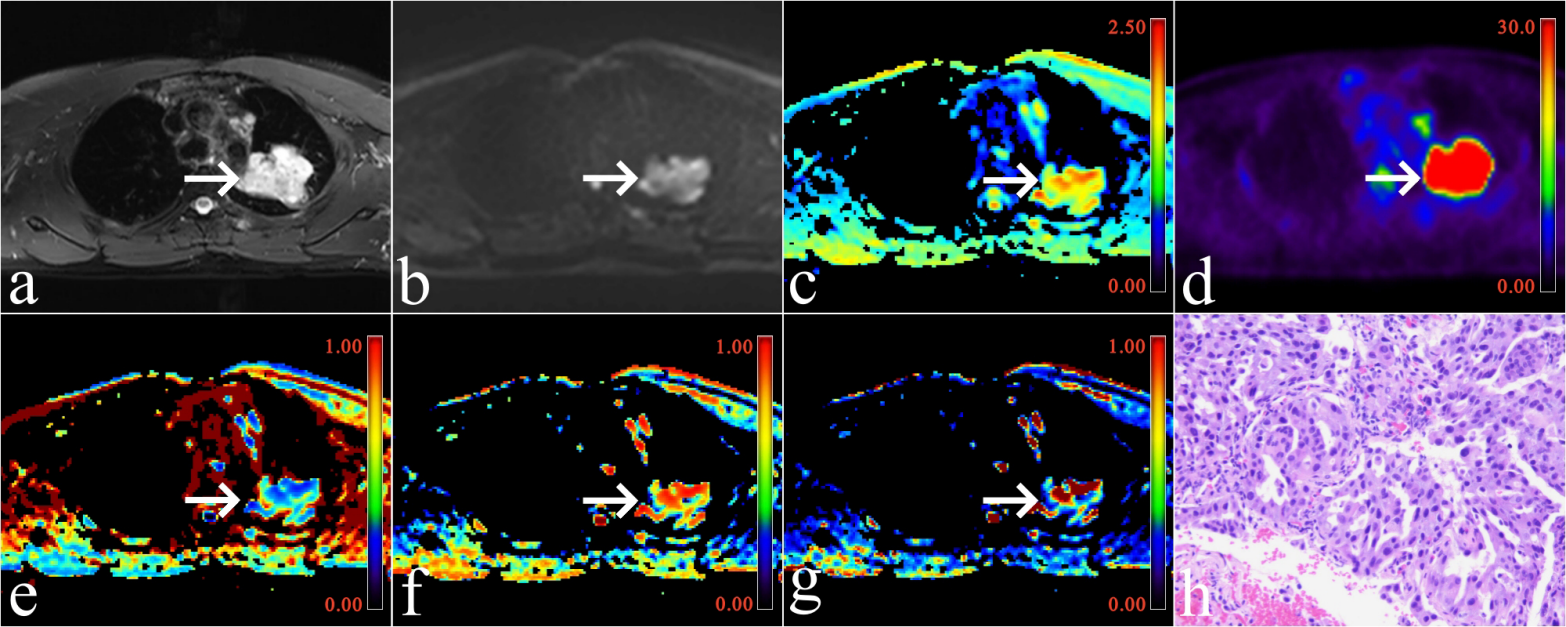
A male patient with adenocarcinoma of the upper lobe of the right lung (arrowheads, maximum diameter = 5.5cm, no smoking). **(a)** Map of T2-weighted imaging; **(b)** Map of DWI (b = 600 s/mm^2^); **(c)** Map of ^18^F-FDG PET; **(d)** Pseudo colored map of ADC; **(e)** Pseudo colored map of f_1_; **(f)** Pseudo colored map of f_2_; **(g)** Pseudo colored map of f_3_; **(h)** Pathological images (original magnification, ×100).

A total of 30 patients with SCC and 67 patients with AC were included. Significant differences were observed between the two groups in maximum lesion diameter (P < 0.001), smoking status (P < 0.001), and sex distribution (P = 0.022). However, there was no significant difference in age between the two groups (P = 0.614). The clinical characteristics are summarized in **Figure 1**, **Table 2**.

**Table 2:** Comparison of various variables between SCC and AC groups.

Variables	SCC (n = 30)	AC (n = 67)	t/χ^2^/z value	P-value
Age (years)	62.47 ± 8.85	61.51 ± 8.01	0.508	0.614^a^
Sex distributionn (%)			5.235	0.022^b^
Female	5 (16.67%)	27 (40.30%)		
Male	25 (83.33%)	40 (59.70%)		
Maximum lesion diameter (cm)	3.45 (2.38, 4.50)	2.00 (1.50, 3.00)	-3.382	< 0.001^c^
Smoking status n (%)	21/30 (70.00%)	21/67 (31.34%)	12.613	< 0.001^b^
f_1_ (×10^−2^)	67.11 ± 17.27	86.27 ± 13.01	-5.426	< 0.001^a^
f_2_ (×10^−2^)	8.71 (1.64, 12.93)	0.24 (0.00, 4.33)	-4.278	< 0.001^c^
f_3_ (×10^−2^)	21.39 (11.88, 33.68)	8.32 (0.01, 19.41)	-4.402	< 0.001^c^
ADC (×10^−3^mm^2^/s)	1.11 ± 0.35	1.37 ± 0.25	-3.600	< 0.001^a^
SUV_max_ (g/cm^3^)	9.93 (5.30, 12.80)	3.77 (1.88, 6.64)	-4.184	< 0.001^c^

AC, Adenocarcinoma; SCC, Squamous Cell Carcinoma.

a Independent t-test.

b Chi-squared test.

c Mann–Whitney U test.

### ICC test

Measurements of f_1_, f_2_, f_3_, ADC, and SUV_max_ showed excellent interobserver agreement, with all ICC values > 0.80. Therefore, the average values from both readers were used for subsequent analysis.

### Parameter comparison

**Figure 2:**
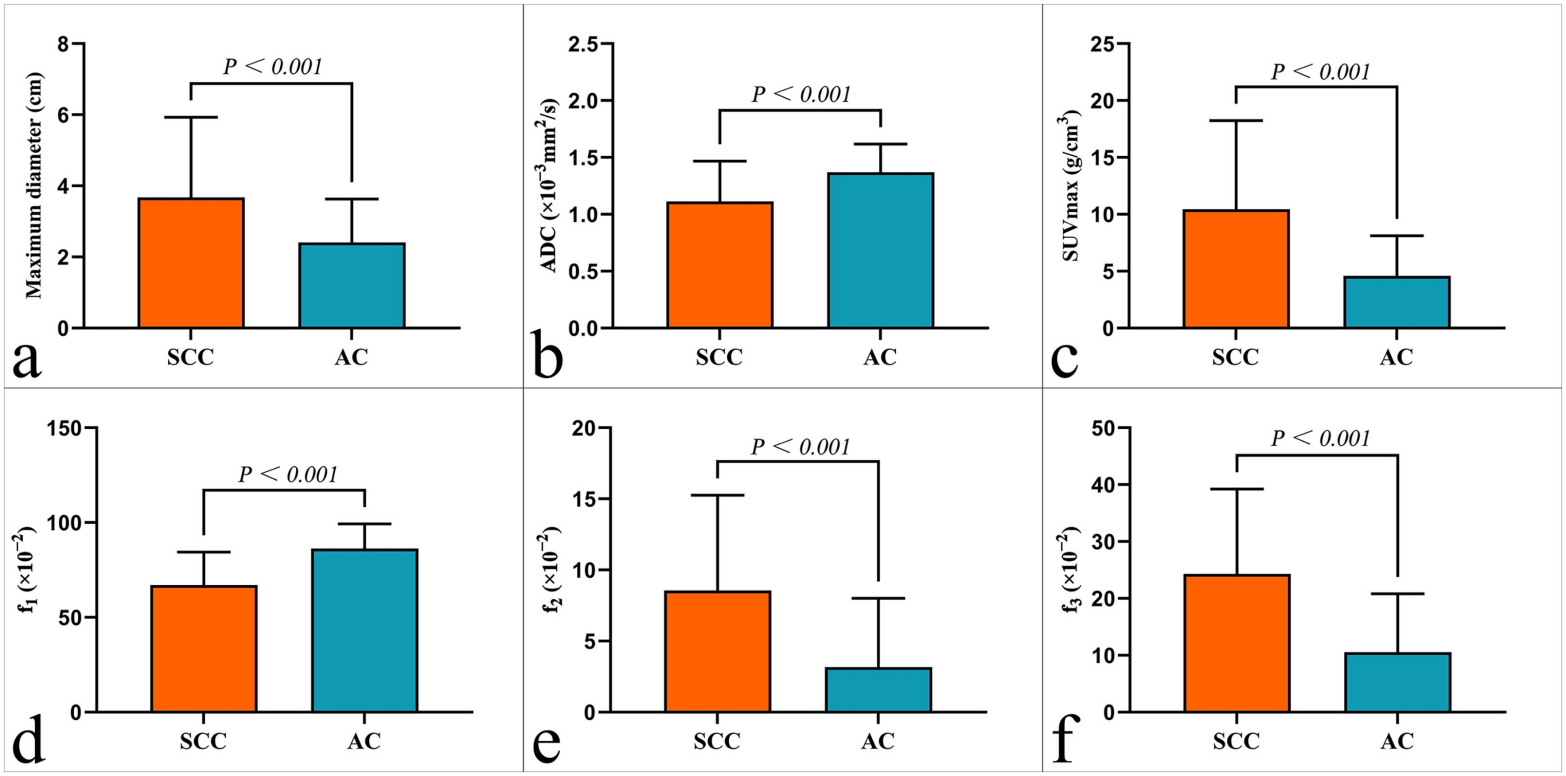
Comparison of **(a)** maximum lesion diameter, **(b)** ADC, **(c)** SUV_max_, **(d)** f_1_, **(e)** f_2_ and **(g)** f_3_ between squamous cell carcinoma (SCC) and adenocarcinoma (AC) groups.

The SCC group exhibited significantly higher SUV_max_, f_2_ and f_3_ values compared to the AC group, while ADC and f_1_ values were lower (all P < 0.05, **Figure 2**, **Table 2**).

### *LR* analysis

Univariate analysis identified sex distribution, smoking status, maximum lesion diameter, f_1_, f_2_, f_3_, ADC, and SUV_max_ as significant predictors for differentiating SCC from AC (all P < 0.05). Multivariate analysis identified smoking status, f_1_, ADC, and SUV_max_ as independent predictors of differentiation, with corresponding P-values of 0.024, 0.001, 0.033, and 0.018, respectively (**Table 3**).

**Table 3:** Univariate and multivariate analyses.

Parameters	Univariate analyses		Multivariate analyses	
	OR (95% CI) *P*-value		OR (95% CI) *P*-value	
Age (year)	1.014 (0.962 ~ 1.069)	0.595	/	**/**
Female	7.407 (2.523 ~ 21.750)	< 0.001	/	**/**
Maximum lesion diameter (cm)	1.709 (1.213 ~ 2.409)	0.002	/	**/**
Smoking	5.111 (2.004 ~ 13.003)	< 0.001	4.266 (1.205 ~ 15.012)	0.024
f_1_ (×10^−2^)	0.920 (0.886 ~ 0.955)	< 0.001	0.918 (0.872 ~ 0.967)	0.001
f_2_ (×10^−2^)	1.171 (1.078 ~ 1.272)	< 0.001	/	/
f_3_ (×10^−2^)	1.094 (1.047 ~ 1.143)	< 0.001	/	/
ADC (×10^−3^mm^2^/s)	0.030 (0.004 ~ 0.241)	< 0.001	0.045 (0.003 ~ 0.781)	0.033
SUV_max_ (g/cm^3^)	1.258 (1.119 ~ 1.414)	< 0.001	1.193 (1.031 ~ 1.380)	0.018

All factors with P < 0.05 in univariate analyses were included in multivariate regression analyses. OR, odds ratio; CI, confidence interval.

### Diagnostic performance

**Figure 3:**
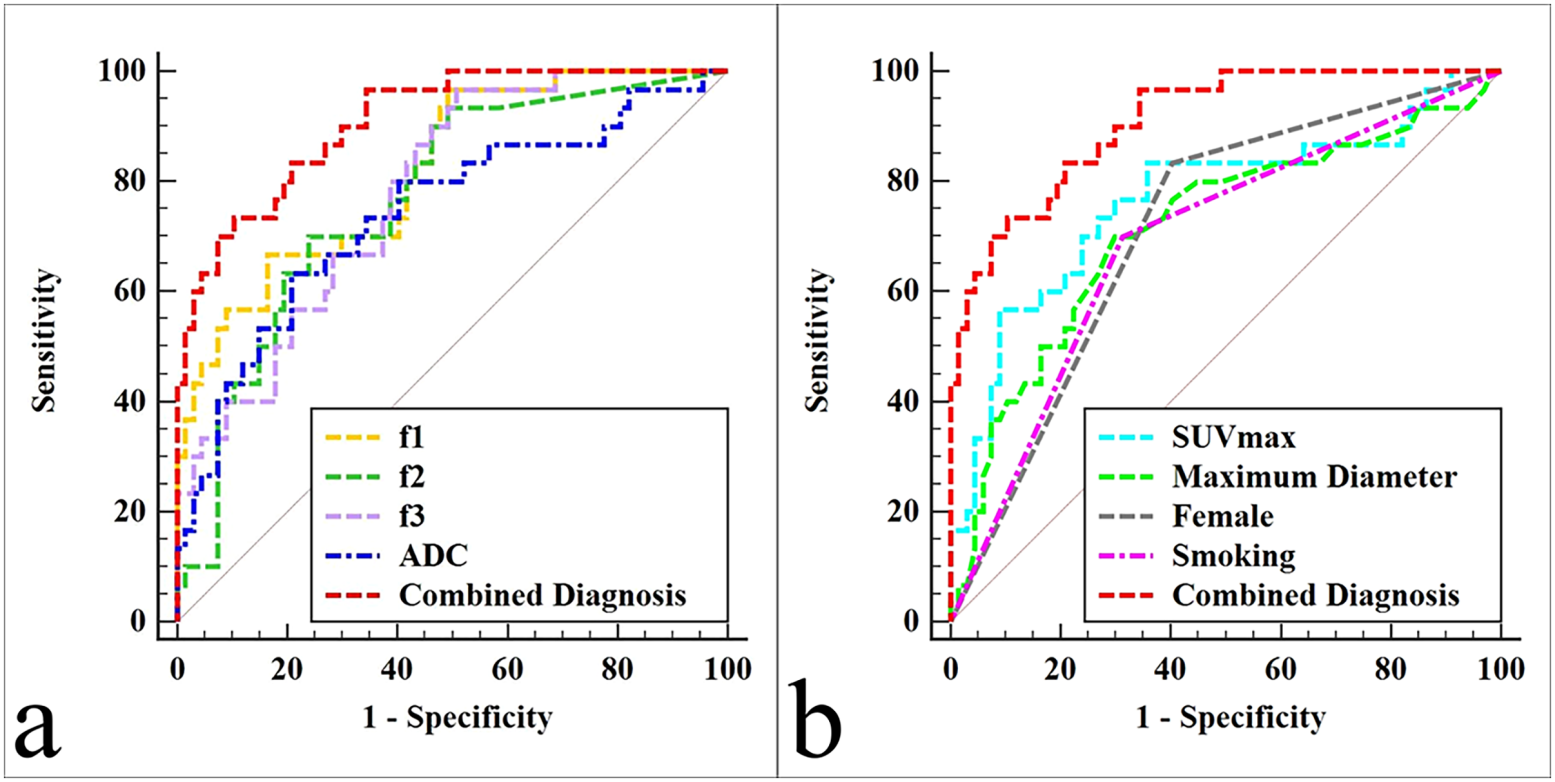
The areas under receiver-operator characteristic (ROC) curves: **(a)** includes lines for f_1_, f_2_, f_3_, ADC, and Combined Diagnosis (smoking + f_1_ + SUV_max_ + ADC); **(b)** includes lines for SUV_max_, Maximum Diameter, Female, Smoking, and Combined Diagnosis (smoking + f_1_ + SUV_max_ + ADC).

The composite model of independent predictors achieved the best diagnostic performance, with an AUC of 0.909, a sensitivity of 73.33%, and a specificity of 89.55%. This performance was significantly superior to that of individual parameters (sex, smoking status, maximum lesion diameter, f_1_, f_2_, f_3_, ADC, and SUV_max_; AUCs = 0.715, 0.693, 0.715, 0.819, 0.769, 0.780, 0.742, and 0.767; Z = 4.175, 4.252, 3.525, 2.252, 2.994, 2.758, 3.194, and 2.960; P < 0.001, < 0.001, < 0.001, = 0.024, = 0.003, = 0.006, = 0.001, and = 0.003, respectively) (**Figure 3**, **Table 4**).

**Table 4:** Predictive performance of different variables.

Variables	AUC (95% CI)	Cutoff	Sensitivity	Specificity	Comparison with a combined diagnosis
Female	0.715 (0.615 ~ 0.802)	/	83.33%	59.70%	Z = 4.175, P < 0.001
Maximum lesion diameter (cm)	0.715 (0.615 ~ 0.802)	2.600	70.00%	70.15%	Z = 3.525, P < 0.001
Smoking	0.693 (0.591 ~ 0.783)	/	70.00%	68.66%	Z = 4.252, P < 0.001
f_1_ (×10^−2^)	0.819 (0.728 ~ 0.890)	71.42	66.67%	83.58%	Z = 2.252, P = 0.024
f_2_ (×10^−2^)	0.769 (0.672 ~ 0.848)	4.329	70.00%	76.12%	Z = 2.994, P = 0.003
f_3_ (×10^−2^)	0.780 (0.685 ~ 0.858)	8.166	96.67%	49.25%	Z = 2.758, P = 0.006
ADC (×10^−3^mm^2^/s)	0.742 (0.643 ~ 0.825)	1.167	63.33%	79.10%	Z = 3.194, P = 0.001
SUV_max_ (g/cm^3^)	0.767 (0.670 ~ 0.847)	9.860	56.67%	91.04%	Z = 2.960, P = 0.003
Combined Diagnosis	0.909 (0.833 ~ 0.958)	0.443	73.33%	89.55%	/

The combined diagnosis implies the combination of independent predictors and represents Smoking + f_1_ + ADC + SUV_max_, CI, confidence interval.

### Validation

**Figure 4:**
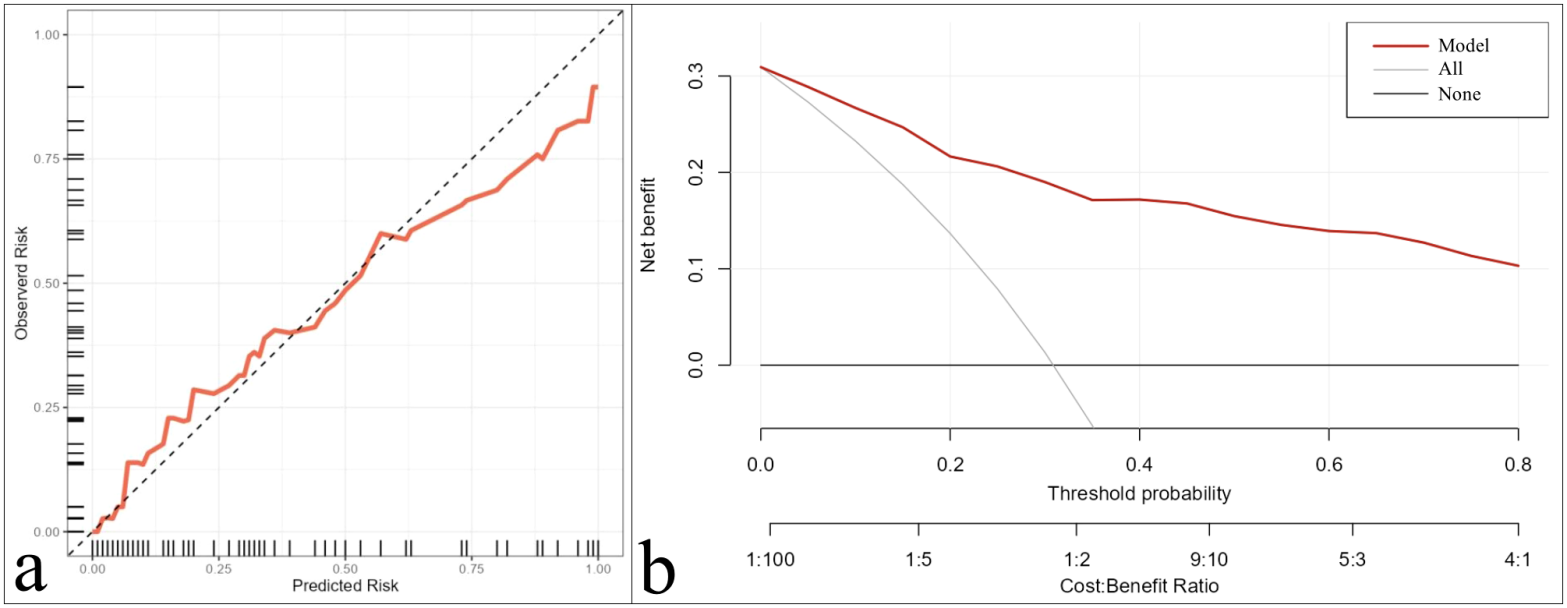
Calibration curve **(a)** and decision curve analysis **(b)** of the combination of independent predictors (smoking + f_1_ + SUV_max_ + ADC).

Internal validation using bootstrap resampling confirmed the robustness of the composite model, yielding an AUC of 0.895 (95% CI: 0.875–0.906). The calibration curve and DCA plots demonstrated good calibration and clinical utility of the model for patients with NSCLC (**Figure 4**).

## Discussion

Currently, ^18^F-FDG PET and DWI are widely used noninvasive imaging modalities for assessing glucose metabolism and water molecule diffusion, respectively. SUV_max_, derived from ^18^F-FDG PET, reflects the peak glucose metabolism of tumors, while ADC, derived from DWI, quantifies the diffusion rate of water within tissues (21). Previous studies have reported that SCC has greater proliferative and invasive potential than AC, leading to distinct ^18^F-FDG metabolism levels and water diffusion rates. This makes SUV_max_ and ADC effective for distinguishing between the two subtypes (22, 23). In this study, SUV_max_ was significantly higher and ADC was lower in the SCC group than in the AC group, which is consistent with previous findings. Moreover, multivariate analysis confirmed that SUV_max_ and ADC are independent predictors, further supporting the utility of ^18^F-FDG PET and DWI in the assessment of NSCLC subtypes.

Building on the aforementioned research, this study innovatively introduced RSI into the differentiation of SCC from AC. RSI is an advanced diffusion imaging technique that assesses the movement of water molecules within human tissues (24). Unlike DWI, RSI does not assume a Gaussian distribution of water diffusion. Instead, it distinguishes and quantifies diffusion across multiple microstructural compartments (restricted, hindered, and free diffusion), allowing for a more precise evaluation of water movement (25). However, clinical studies on RSI have yielded inconsistent findings. For example, in breast cancer, both f_1_ and f_3_ can differentiate benign from malignant lesions, with malignant lesions exhibiting increased f_1_ and decreased f_3_ values (26). In contrast, in rectal cancer, only f_1_ can effectively distinguish high-grade from low-grade tumors, with higher f_1_ values observed in high-grade cases (27). In the present study, compared with the AC group, the SCC group exhibited lower f_1_ values and higher f_2_ and f_3_ values. Among these parameters, f_1_ was identified as an independent predictor for distinguishing SCC from AC. The parameters f_1_, f_2_, and f_3_ represent the proportions of restricted, hindered, and free diffusion, respectively, and their sum equals 1 (10). A possible explanation for the observed differences lies in the biological characteristics of the two tumor types. Although SCC is typically more invasive and has a higher cell density than AC (which would suggest an increase in the restricted diffusion compartment) (28, 29), the associated tissue microischemia and micronecrosis may expand the extracellular space and increase the free water content. This shift promotes greater hindered and free water diffusion. When the increase in restricted diffusion is outweighed by the increase in hindered and free diffusion, the f_1_ fraction decreases, leading to elevated f_2_ and f_3_ values (30). Additionally, differences in cell arrangement between SCC and AC may also contribute to this outcome.

Clinical factors, including age, sex distribution, smoking status, and maximum lesion diameter, were incorporated into the analysis. The results indicated that while sex distribution, smoking status, and maximum lesion diameter contributed to the differentiation of SCC from AC, only smoking status emerged as an independent predictor. This finding aligns with previous studies, reinforcing the role of smoking status as a simple and effective indicator for NSCLC subtyping (31). The underlying mechanism may be attributed to smoking-induced bronchial squamous epithelial carcinogenesis (32).

Despite these promising results, several limitations must be acknowledged. First, this was a single-center study with a relatively small sample size and an uneven distribution of tumor subtypes (30 cases of SCC vs. 67 cases of AC), which may affect the stability and generalizability of the predictive model. Second, research on RSI sequences—particularly in lung imaging—is still limited, and the optimal b-value for lesion evaluation has not yet been established. Third, MRI has limitations in detecting microscopic lesions. Fourth, despite the use of various techniques to mitigate respiratory and cardiovascular pulsation artifacts, their impact remains significant. Fifth, previous studies have suggested an association between tumour location and subtypes of NSCLC. Among the clinical factors considered in this paper, lesion location was not included as a potential influencing factor, which could have an adverse impact on the research results. Future research will focus on expanding sample sizes, conducting multicenter studies, reducing distribution disparities among different lesion subtypes, and incorporating additional clinical factors such as lesion location. Additionally, efforts will be made to optimize scanning protocols and improve image quality to ensure more stable and reliable results.

## Conclusion

Smoking status, f_1_, SUV_max_, and ADC are independent predictors for the differentiation of AC from SCC. The combination of these parameters shows potential as a biomarker for the classification of NSCLC subtypes.

## Data Availability

The raw data supporting the conclusions of this article will be made available by the authors, without undue reservation.
